# Acknowledgement to Reviewers of *Polymers* in 2015

**DOI:** 10.3390/polym8010025

**Published:** 2016-01-21

**Authors:** Polymers Editorial Office

**Affiliations:** MDPI AG, Klybeckstrasse 64, CH-4057 Basel, Switzerland; polymers@mdpi.com

The editors of *Polymers* would like to express their sincere gratitude to the following reviewers for assessing manuscripts in 2015.

We greatly appreciate the contribution of expert reviewers, which is crucial to the journal’s editorial decision-making process. Several steps have been taken in 2015 to thank and acknowledge reviewers. Good, timely reviews are rewarded with a discount off their next MDPI publication. By creating an account on the submission system, reviewers can access details of their past reviews, see the comments of other reviewers, and download a letter of acknowledgement for their records. In addition, MDPI has launched a collaboration with Publons, a website that seeks to publicly acknowledge reviewers on a per journal basis. This is all done, of course, within the constraints of reviewer confidentiality. Feedback from reviewers shows that most see their task as a voluntary and mostly unseen work in service to the scientific community. We are grateful to our reviewers for the contribution they make.

Affolter, ChristianHong, JinkeePatrickios, CostasAgo, MarikoHorrocks, RichardPauer, WernerAhmadiannamini, PejmanHorsch, MartinPegoretti, AlessandroAhn, Kyung HyunHossain, Khandaker M. A.Pérez-Murano, FrancescAkhlaghi Amiri, Hossein A.Hossain, M. E.Persano, LuanaAkitsu, TakashiroHowell, BobPeter, MartinAl-Haik, MarwanHowlader, M. M. R.Pettersson, TorbjörnAl-Mosawe, AlaaHowlin, BrendanPham, Viet HungAli, Shaikh A.Hozumi, AtsushiPigge, F. ChristopherAltarawneh, MohammednoorHsiao, Pai-YiPignatello, RosarioAltmann, BrigitteHu, MengPiletsky, SergeyAlvarado, VladimirHuang, WeiminPina, SandraAmeri, TayebehHuang, Chun JenPinho, EvaAmiri, AliHuang, JianweiPintauer, TomislavAmo-Ochoa, PilarHüffer, ThorstenPinto, JavierAndrieu-Brunsen, AnnetteHutchinson, Robin A.Pissis, PolycarposAnthamatten, MitchellHwang, Do-HoonPlamper, FelixAntipina, Maria N.Hwang, NathanielPochat-Bohatier, C.Antunes, MarceloHyun, JinhoPoli, RinaldoAoki, TakashiIatrou, HermisPons, JosefinaApaydin, K.Ie, YutakaPopescu, CosminArbelaiz, AitorIm, Seung SoonPopov, Anatoliy V.Asatekin, AyseImaz, InharPoriel, CyrilAshraf, ShahidImbrogno, AlessandraPotirniche, GabrielAvarvari, NarcisIqbal, SamirPouponneau, PierreAvgeropoulos, ApostolosIraqi, AhmedPoverenov, ElenaBabaeidarabad, SamanIshikawa, ToshiyukiPragliola, StefaniaBachmann, MichaelIshikawa, YuichiPrice, Richard B. T.Backer, HansIwata, RintaroPriftis, DimitrisBadogiannis, E.Jabbari, MostafaPucci, AndreaBae, Young ChanJablonski, Monica M.Punzo, FrancescoBai, JinboJaksch, SebastianQiao, KunBaino, FrancescoJanát-Amsbury, Margit M.Quaglia, FabianaBakis, CharlesJassby, DavidQuintana, Ainhoa LucíaBalogun, OlaniyiJelliss, Paul A.Quirino, RafaelBaran, DeryaJeong, Han-SolRaabe, DirkBaranoff, EtienneJeong, Kwang CheolRadusch, Hans-JoachimBarkoula, N. -M.Jhurry, DhanjayRaftoyiannis, Ioannis G.Bashur, ChristopherJordi Aguilera-Sigalat, JordiRahaman, SaifurBattaglia, LuigiJung, In HwaRahier, HubertBebawy, MenaJung, Young MeeRahimabady, MojtabaBella, FedericoJunkers, ThomasRajan, VarunBender, HugoKakroodi, Adel RamezaniRam, Manoj K.Benyahia, BrahimKalantar-zadeha, KouroshRamineni, SandeepBergbreiter, David E.kang, Sang WookRana, D.Bisht, Gobind SinghKanjwal, Muzafar A.Rao, A.Boateng, Joshua S.Kantzas, ApostolosRasoul, Firas A.Bonnet, FannyKaralekas, DimitrisRaucci, Maria GraziaBorges, JoãoKavalenka, Maryna N.Rawls, HenryBorriello, AnnaKelnar, I.Reis, JoaoBosch, P.Kenneth, DollRibeiro, AntonioBourissou, DidierKhan, AnzarRicco, PierreBournas, DionysiosKhan, Omar F.Richaud, EmmanuelBoyer, CyrilleKim, Hee-TakRiedl, BernardBreveglieri, MatteoKim, Young HoonRivas, Bernabé L.Bronstein, HugoKim, Sang-JaeRodrigues, Debora F.Caliceti, PaoloKim, Sung WanRonca, S.Camões, A.Kim, Soo YoungRousakis, TheodorosCamposeo, AndreaKim, Ill WonRuan, JrjengCapacchione, CarmineKim, Cheol SangRubenstein, David A.Carpentier, Jean-FrançoisKimura, YoshiharuRuggieri, FabrizioCarraro, MauroKitamoto, Hiroko KuzeRussell, GregoryCasas, Joan R.Knez, MatoRussell, Thomas P.Castillo, OscarKobayashi, KoichiRusso, PietroCavaco-Paulo, ArturKobayashi, ShingoSaito, HiromuChen, C. WillKoike, KotaroSakaguchi, ToshikazuChen, Bor-kuanKok, R. J.Salerno, AurelioChen, Mei-HsinKoller, MartinSamper, M. D.Chen, AnKontou, E.Sanna, VannaChen, Ching-YiKoschella, AndreasSannino, DianaChen, Cheng-LungKotobuki, MasashiSantulli, C.Cheng, XingguoKowalczuk, AgnieszkaSarasua, Jose-RamonCheng, YilongKratz, KarlSasan, NouranianChiappone, AnnalisaKrifa, MouradSatoh, ToshifumiChiu, Tai-chiaKuang, HuihuiSauve, GenevieveCho, Seung HeiKubo, TakuyaSaxena, TarunCho, Hyun-JongKuckling, DirkScaffaro, RobertoCho, Dong-WooKuik, MartijnScalet, G.Choi, PangilKuktaite, RamuneScarfato, PaolaChoi, Hyoung JinKumar, SandeepSchartel, BernhardChou, JoshuaKumar, Srinivasan DineshSchenning, Albertus P. H. J.Chuang, John Shih-ChingKuo, Shiao-WeiSchlettwein, DerckCladera, AntoniKuo, Dong-HauSchröder, HendrikClarke, AnthonyKwon, Jung-HwanSchroeder, BobCole, Marsha R.La Mantia, Francesco PaoloSeidel, Rüdiger W.Colò, FrancescaLadavos, AthanasiosSena-Cruz, JoseColombi, PierluigiLai, Jui-yangSeo, Dae-ShikColombo, AlessiaLaurienzo, PaolaSeo, YongsokCong, WeilongLavina, SandraSerra, AngelsConnal, Luke A.Lavrentovich, Oleg D.Sevink, G. J. AgurContreras-Cáceres, RafaelLázár, IstvánSheiko, Sergei S.Cook, Wayne D.Leadbeater, NicholasShendruk, TylerCorbi, OttaviaLecomte, PhilippeShiau, Bor-jierCorbi, IleanaLee, Beom-JinShibasaki, YujiCordier, StéphaneLee, ChiSidorenko, AlexanderCorreia, LuísLee, Chang-ChunSikorski, Pawel TadeuszCosta, RuiLee, Bruce P.Silva, M. M.Covas, J. A.Lee, Hyung IlSilvennoinen, RaimoCroce, FaustoLee, Bor-ShiunnSimsek, ErenCroll, Stuart G.Lee, Wen-FuSkirtach, AndreCrook, VincentLee, Hyung KeunSloop, JosephCrouzier, ThomasLee, Jin WooSoares, Edson J.Cudziło, StanisławLeone, G.Sotta, PaulCui, HaitaoLeporatti, StefanoSpadea, GiuseppeD'hooge, DagmarLi, QingfengSpirk, StefanDatta, J.Li, SumingSreekumar, P. A.David, LaurentLi, MingqiangSrinivasan, M. P.Davids, BillLi, GuoqiangStasch, AndreasDe Brito, JorgeLignola, Gian PieroStieglitz, ThomasDeifalla, A.Lim, Kwon TaekStreet, RobertDel Teso Sánchez, KarmeleLim, JeewooSu, Chean-ChengDel Vecchio, CiroLin, Hung-YinSu, Wen-DaDelferro, MassimilianoLindén, MikaSubramanian, ChitrabalaDelgado, AngelLionetto, FrancescaSuckeveriene, RanDeligianni, D.Liotta, Charles L.Sugawara-Narutaki, AyaeDelongchamp, DeanLiu, SongSundman, OlaDeng, YongmingLiu, Shou-HengSuryanto, BennyDervisi, AthanasiaLiu, RuiSwindle-Reilly, Katelyn E.Di Ludovico, MarcoLiu, YeTabe, ShahramDi Maio, LucianoLiu, YinTakashima, KazutoDias, Rolando C. S.Lodge, Timothy P.Takenaka, MikihitoDias, JulianaLopez-Buendia, Angel MTanaka, ManabuDo, ChangwooLopez-Sanchez, PatriciaTanaka, KazuoDomenek, SandraLotti, NadiaTang, BingtaoDomenici, ValentinaLou, TiejiongTannert, ThomasDöring, ManfredLow, Bee TingTao, LeiDragoni, EugenioLuo, JuntaoTappan, Bryce C.Drioli, EnricoLuo, JiangshuiTaurin, SebastienDrummond, CarlosLuong, Dzung DinhTedim, JoãoDulong, VirginieLuscombe, ChristineTeramoto, HidetoshiDuong, Hai M.Lyon, R. E.Thota, SammaiahDzubiella, JoachimMaas, M.Tiainen, HannaEbara, MitsuhiroMaazouz, AbderrahimTobita, HidetakaEe, Pui-Lai RachelMacDonald, MelissaToda, AkihikoEguiazábal, José I.Maciejewska, DorotaTokita, MasatoshiElbert, Donald L.Magalhães, JoanaTomlinson, DouglasElliott, Gloria D.Magda, JulesTonge, MatthewEndo, TakeshiMagnusson, Johannes PallTorrens, FranciscoEsnouf, StéphaneMai, WenjieTorres-Lagares, DanielEstellé, PatriceMarder, Todd B.Tran, PhuongEthirajan, AnithaMariani, AlbertoTsai, HsinyuFally, MartinMaric, MilanTsarevsky, N.Fambri, LucaMarrocchi, AssuntaTsuji, HidetoFaruqi, Mohamed A.Massue, JulienTsujimoto, TakashiFernandes, SuseteMatsumoto, AkikazuTuba, RobertFernandes, Susana C. M.Matsuo, TakashiTyrode, EricFerrier, E.Mavrantzas, VlasisUnterreiner, Andreas-NeilFilippov, SergeyMawhinney, Thomas P.Valiyaveettil, SureshFischer, DagmarMbey, J.A.Van Herk, AlexFocacci, FrancescoMcAnulty, JonathanVanderzande, Dirk J. M.Foged, CamillaMei, YingVautard, FredericFollain, NadègeMeins, Jean-François LeVeleirinho, BeatrizFossum, EricMeredith, J. CarsonVenkataraman, ShrinivasFoti, DoraMerino, SoniaVenkatesan, JayachandranFrontera, AntonioMerlitz, HolgerVenugopal, GunasekaranFujioka, TakahiroMessori, MassimoVerbeek, JohanFukui, ToshiakiMi, Fwu-LongVerduzco, RafaelFurusawa, HiroyukiMiàs, C.Vignolle, JoanGagliardi, MariacristinaMichels, JulienVilaplana, F.Gao, W. Y.Miner, Jeffrey H.Vlachopoulos, JhonGarcía, AntonioMiosge, NicolaiWagner, Diane R.Garcia, IñakiMischnick, PetraWang, Yu-zhongGeiß, Paul LudwigMisra, DeveshWang, Hsing-LinGerasimchuk, NikolayMiyata, TakashiWang, YangyangGervais, ClaireMoad, GraemeWang, De-YiGhafoori, ElyasMoradian-Oldak, JanetWang, YadongGhrib, FaouziMorgan, AlexanderWang, Tzu-WeiGiménez-Marqués, MónicaMorozov, Victor N.Wang, LiangGoglio, L.Mottram, James TobyWee, Lik H.Gohs, UweMujika, FaustinoWeiss, Clemens K.Gomez, EliotMüller-Buschbaum, PeterWhittaker, Michael R.Gomez, Enrique D.Muzzarelli, Riccardo A. A.Wilén, Carl-EricGomez, AlessandroMyc, AndrzejWillerth, Stephanie MGong, XiongNagao, DaisukeWilliams, PhilGonzález-Rodríguez, María-LuisaNair, MridulaWischke, ChristianGopalan, PadmaNakada, HiroshiWitkowski, ArturGorga, Russell E.Nakamura, Y.Won, Jong-PilGranville, Anthony M.Nakano, KojiWoo, EamorGrassi, GabrieleNart, J.Wu, XiaoyiGregorczyk, KeithNaser, JamalWu, Zi LiangGribniak, ViktorNazari, AliWu, BingGriffini, GianmarcoNejati, SiamakWujcik, EvanGrisi, FabiaNganga, SaraXavier, JoséGu, YizhuoNoël, MartinXu, YunjunGuelcher, Scott A.Notta-Cuvier, DelphineYamoto, TakehikoGuiver, Michael D.Novakovic, KatarinaYan, LiboGundogdu, KenanNulwala, HunaidYang, Po-ChihGuo, JohnNyström, Andreas M.Yang, ZhijunGuo, HuiminO’Connor, Naphtali A.Yeboah, DavidGutekunst, Will R.Oaki, YuyaYeon, Jung HeumGutiérrez Muñoz, CristinaOh, Jae-MinYi, ShouliangHaaparanta, Anne-MarieOhara, HitomiYin, JieHadjiargyrou, MichaelOliva, LeoneYouk, Ji HoHager, Martin D.Olsson, GustafYousefi, Azizeh-MitraHajizadeh, ElnazOmbres, LucianoYousif, BelalHallouard, FrançoisOppelt, Kerstin T.Youssef, Ahmed A.Haraldsson, TommyOrbey, NeseYu, LupingHarding, IanOrtenzi, Marco A.Yu, WeiHarmandaris, VagelisOzbakkaloglu, TogayYu, GuihuaHayward, Ryan C.Ozkul, HulusiYuan, JiayinHe, Wei-dongPan, JinlongZaslavsky, BorisHe, XuehaoPandey, G. P.Zeugolis, DimitriosHe, DongshengPandis, ChristosZhang, QichunHeeney, MartinPang, ChanghyunZhang, JieHeikkinen, Susanna L.Panizza, MatteoZhang, HuHerzing, AndrewPaolone, AnnalisaZhao, Xing-zhongHickey, OwenPapadopoulos, PeriklisZhao, HanyingHidalgo, Javier GarcíaPapageorgiou, George Z.Zhao, YufengHigashi, N.Park, Sung YoungZhu, HaijinHinrichs, Wouter L.J.Park, Chan-GiZiaee, ZainabHirao, AkiraPark, Pyung-KyuZifferer, GerhardHo, JohnnyPark, Chan HeeZikry, Mohammed A.Hodge, PhilipParnell, Andrew J.Zoccola, MarinaHong, Jin-WhoParthasarathy, RajarathinamZuber, GuyHong, HaipingPassalacqua, AlbertoZydney, Andrew L.

